# PREDICTORS OF CONCOMITANT COGNITIVE AND PHYSICAL FUNCTION DECLINE: RESULTS FROM THE HEALTH ABC STUDY

**DOI:** 10.1093/geroni/igz038.303

**Published:** 2019-11-08

**Authors:** Elizabeth Handing, Iris Leng, Kathleen Hayden, Caterina Rosano, Stephen Kritchevsky

**Affiliations:** 1 Wake Forest School of Medicine, Winston-Salem, North Carolina, United States; 2 University of Pittsburgh, PIttsburgh, Pennsylvania, United States

## Abstract

Although many individual risk factors for cognitive and physical decline have been identified, less is known about factors that predict concomitant or “dual decline”. Utilizing data from the Health ABC study (n=2,553), we examined trajectories of decline based on repeated measures of the Modified Mini-Mental State Exam (3MS) and Short Physical Performance Battery (SPPB) across 5 years. Next, we calculated four mutually exclusive trajectories of decline derived from the slope: no decline (change in slope ≥ 0, n= 1,190), cognitive decline (lowest quartile of slope in 3MS; n=445), physical decline (lowest quartile of slope in SPPB; n= 407), and dual decline (lowest quartile for both cognitive and physical decline; n= 211). Logistic regression tested the association of 23 baseline risk factors with dual decline. Odds of dual decline where higher in relation with depression (CES-D >16) (Odds Ratio [OR]: OR:2.77, 95% Confidence Interval [95% CI] [1.25- 6.15]), and lower for individuals with a better score on the Digit Symbol Substitution Test (DSST) (OR: 0.97 [0.95-0.98]), grip strength (OR: 0.97 [0.94-1.00]), and 400m gait speed (OR: 0.11 [0.03-0.40]). Among the predictors identified, depressive mood at baseline nearly tripled the odds of developing dual decline, but was not associated with decline in either the cognitive or physical domains. Depressive mood is uniquely associated with dual decline; future studies should focus on the overlap between mood states and function, as mood may be modifiable.

